# Supplementary material for: Host genome and bacterial taxa shape the Arabidopsis seed microbiome
Source: EMBO Rep. 2025 Nov 28;27(1):122–41. doi: 10.1038/s44319-025-00635-x (PMC12796167; doi:10.1038/s44319-025-00635-x)
Supplement: Supplementary file 1 — Appendix [file 44319_2025_635_MOESM1_ESM.pdf]

Appendix

Table of Contents

Appendix Figure S1……………………………………………………………………………2

Appendix Figure S2……………………………………………………………………………3

Europe: Fe distribution

A.

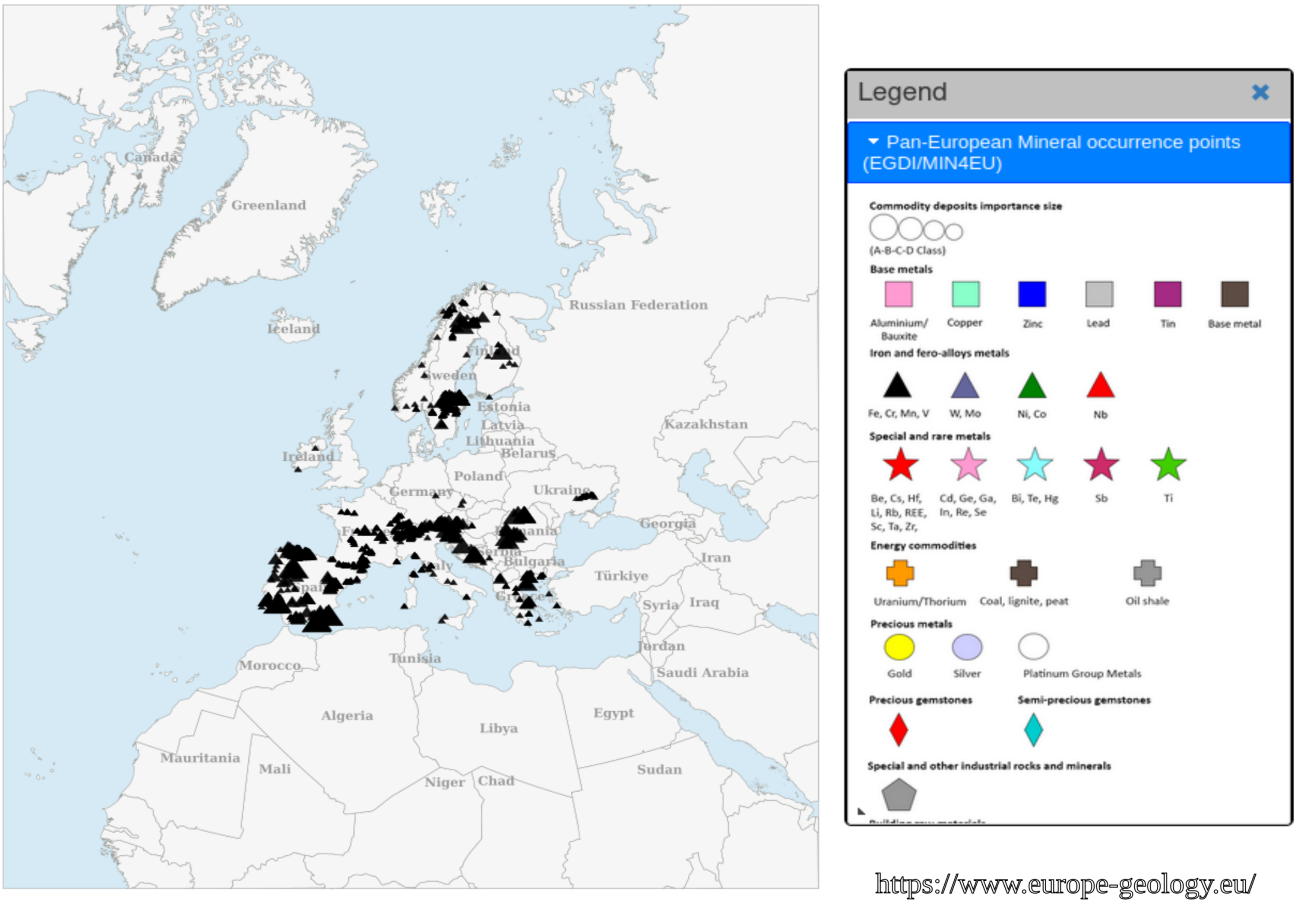

https://www.europe-geology.eu/

Europe: pH distribution

B.

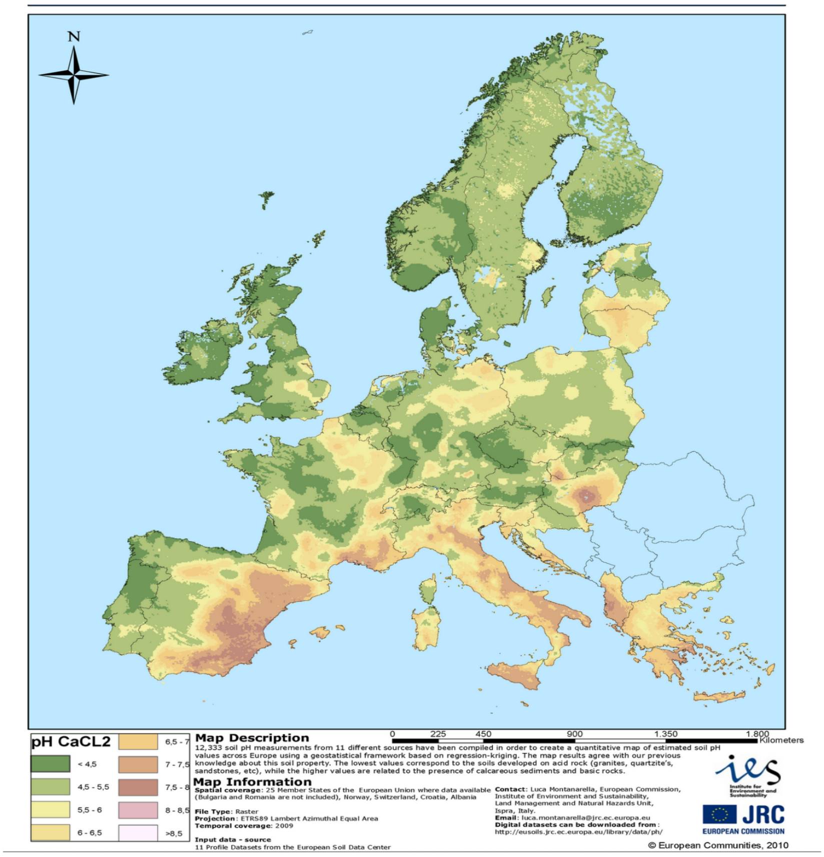

Appendix Figure S1: The distribution of Iron (Fe) and Ph across the Europe region. In (A) the black filled solid triangles show iron with sizes corresponding to concentration. (B) the pH scale across the regions is marked as a heatmap gradient.

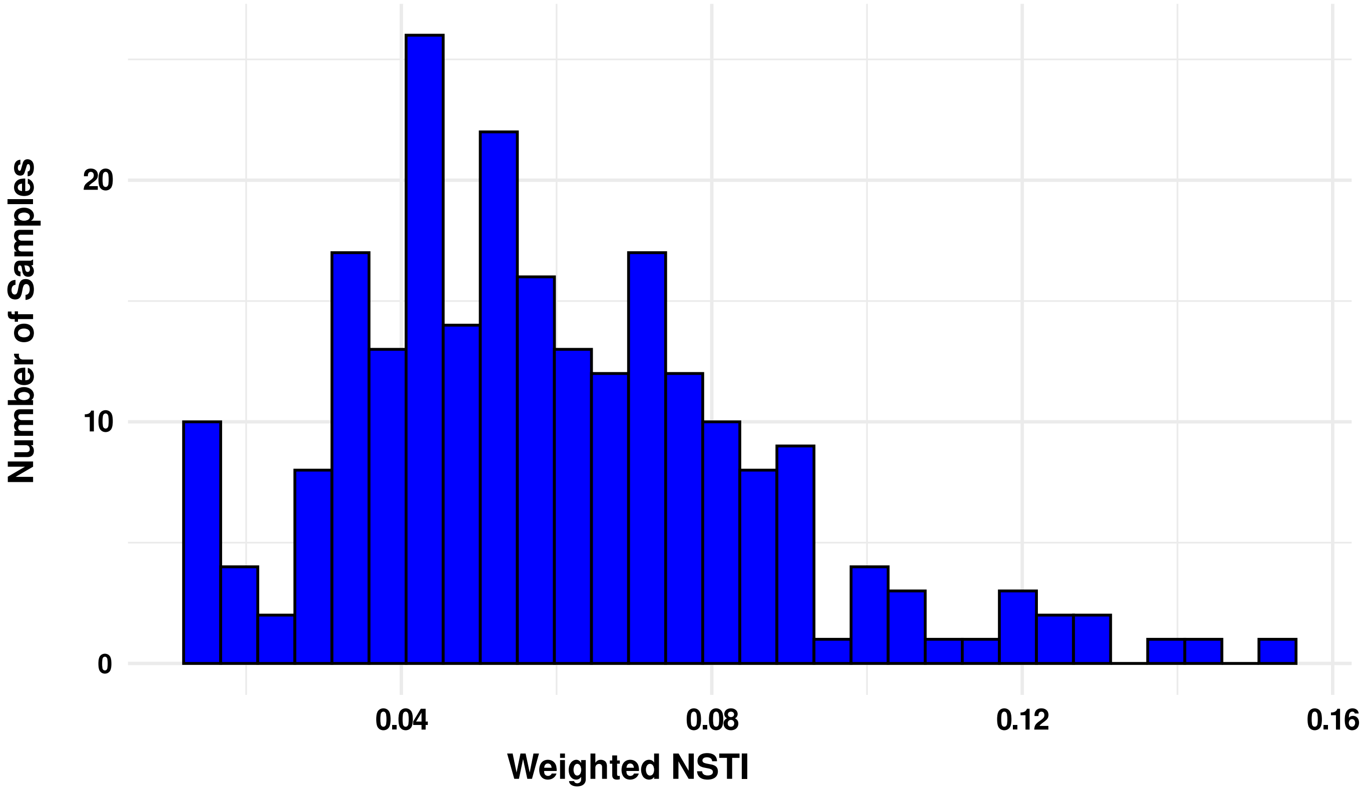

Appendix Figure S2: Distribution of Weighted NSTI values across samples.
Histogram showing the distribution of Weighted Nearest Sequenced Taxon Index (NSTI) values for all analyzed samples. The x-axis represents Weighted NSTI scores, while the y-axis shows the number of samples. Lower NSTI values indicate a closer match between observed amplicon sequence variants (ASVs) and reference genomes in the PICRUSt2 database, reflecting higher confidence in functional predictions. The overall distribution demonstrates that the majority of samples exhibit low to moderate NSTI scores, supporting the reliability of functional inference across the dataset.
