# Supplementary figures and images for: Host genome and bacterial taxa shape the Arabidopsis seed microbiome
Source: EMBO Rep. 2025 Nov 28;27(1):122–41. doi: 10.1038/s44319-025-00635-x (PMC12796167; doi:10.1038/s44319-025-00635-x)

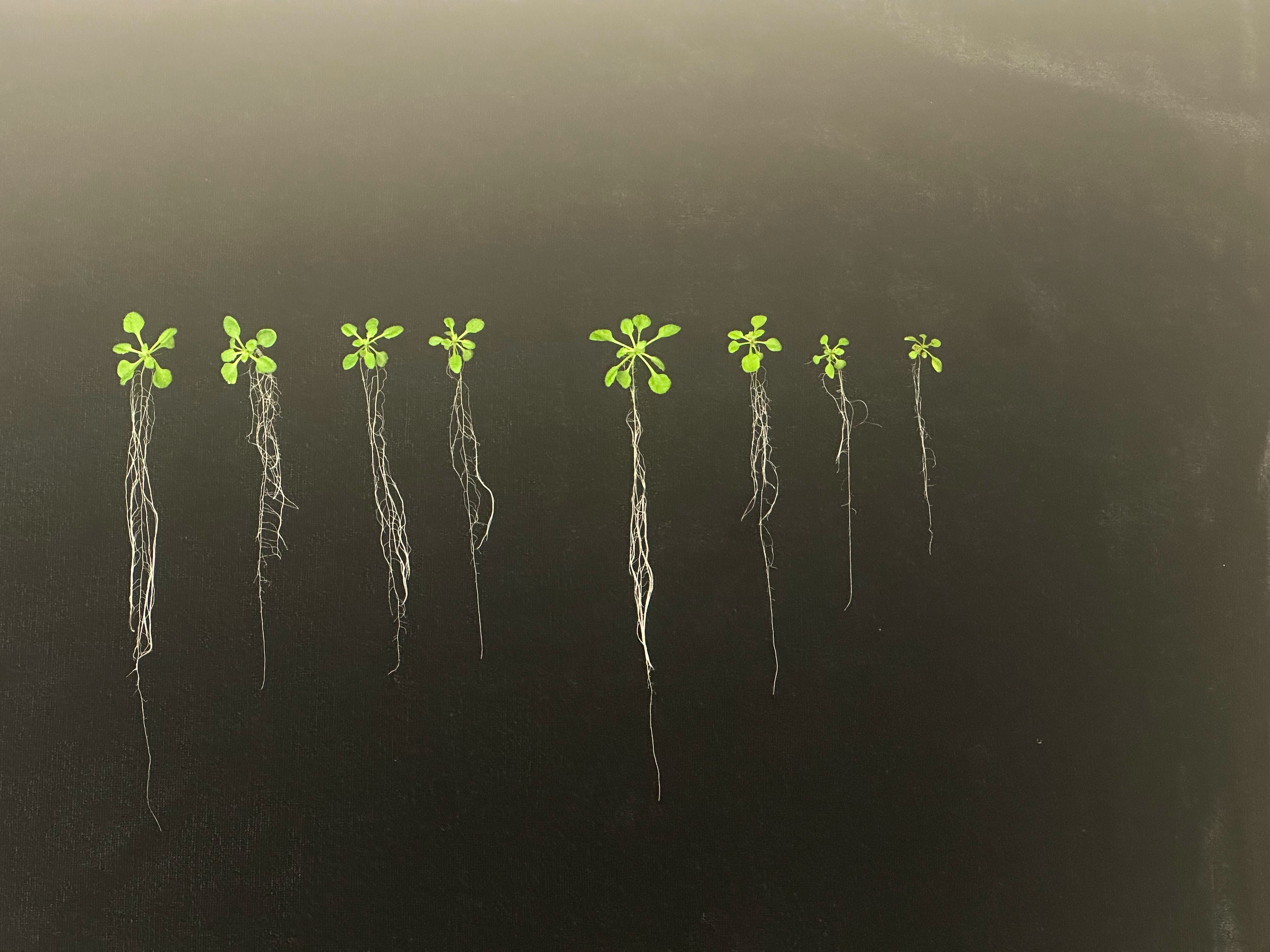

Supplement: Supplementary file 14 — Source data Fig. 6 [file 44319_2025_635_MOESM14_ESM.zip › Figure6/1B/IMG_3264.tif]

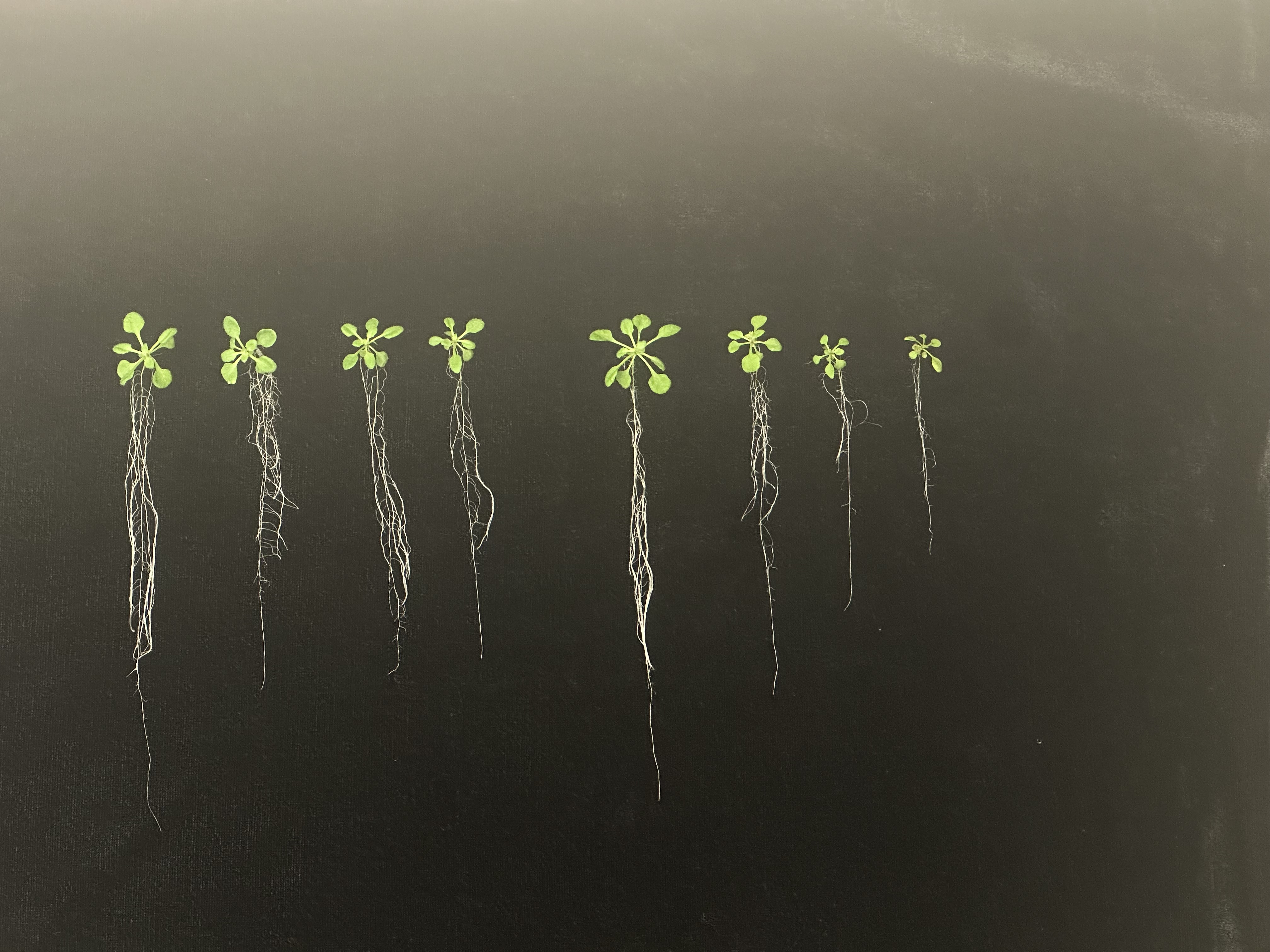

Supplement: Supplementary file 14 — Source data Fig. 6 [file 44319_2025_635_MOESM14_ESM.zip › Figure6/1C/1C.tiff]
