# Supplementary material for: Host genome and bacterial taxa shape the Arabidopsis seed microbiome
Source: EMBO Rep. 2025 Nov 28;27(1):122–41. doi: 10.1038/s44319-025-00635-x (PMC12796167; doi:10.1038/s44319-025-00635-x)
Supplement: Supplementary file 15 — Expanded View Figures [file 44319_2025_635_MOESM15_ESM.pdf]

Expanded View Figures

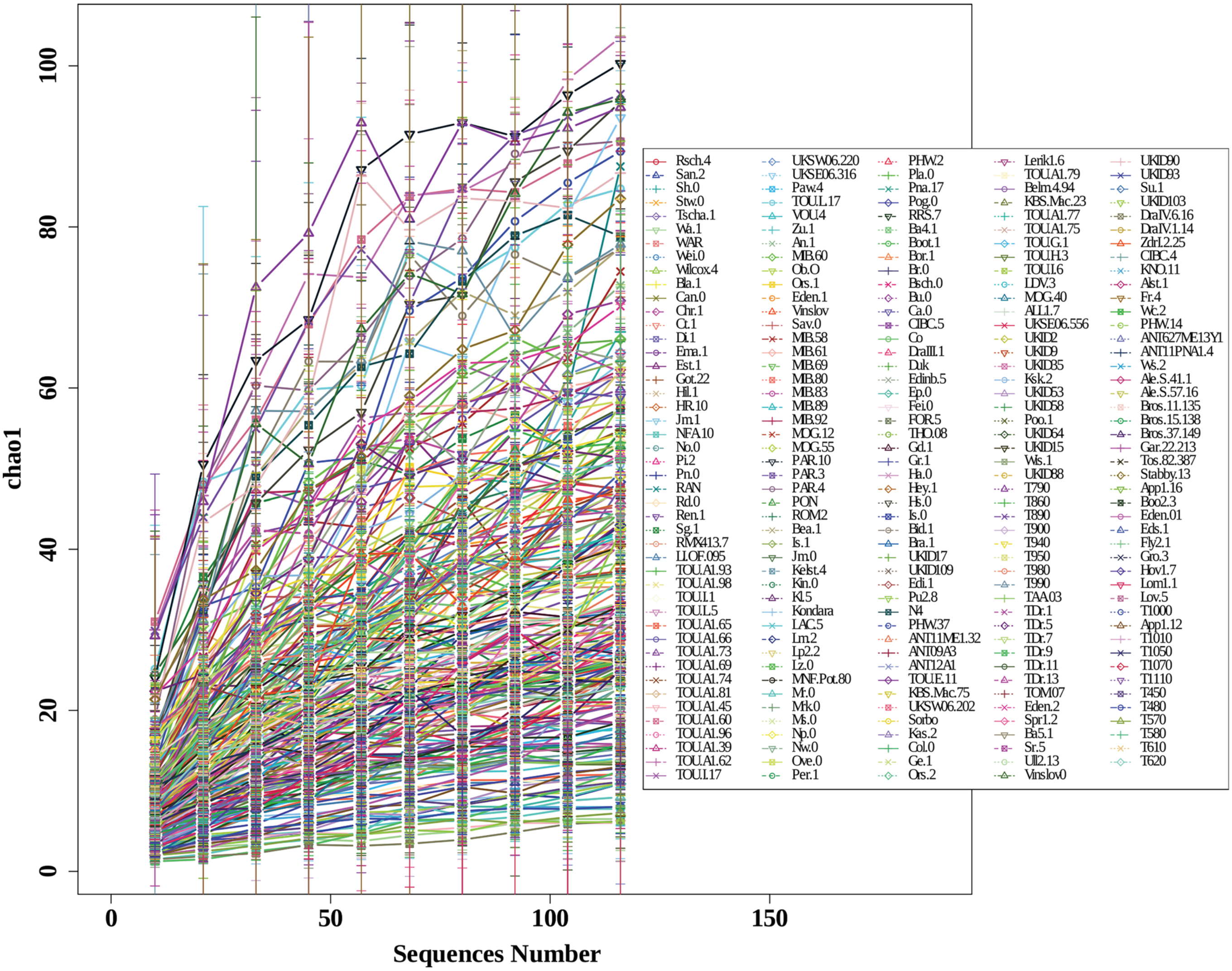

Figure EV1. Rarefaction curve across the accessions.

The richness measured by Chao1 across all the accessions is plotted over the sequence numbers. Each accession is highlighted by a different color in the plot. The curves show that species richness increases with sequencing effort, and several samples approach a plateau, suggesting that their microbial communities have been adequately sampled.

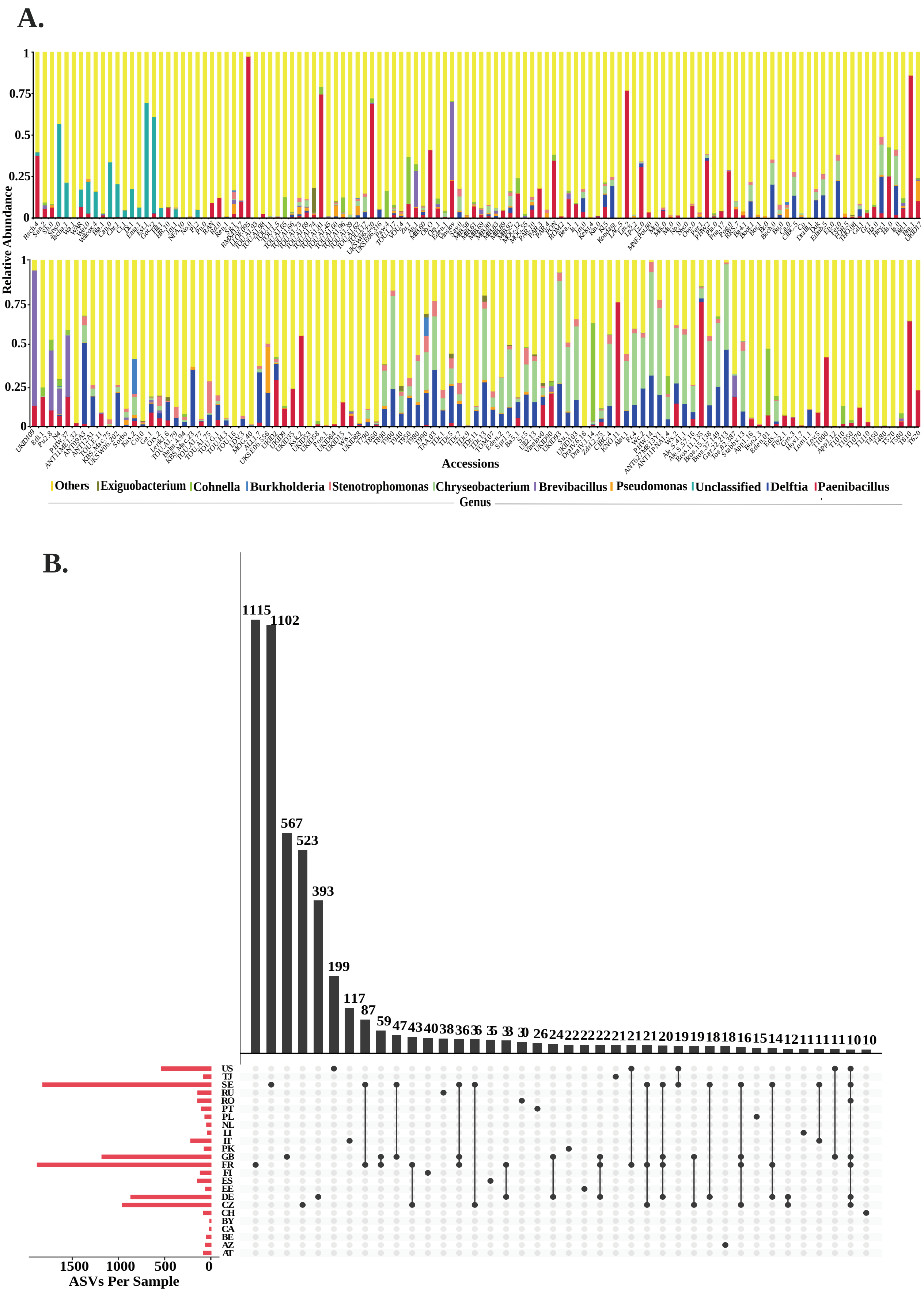
A.
Relative Abundance
1
0.75
0.5
0.25
0
Accessions
Others
Exiguobacterium
Cohnella
Burkholderia
Stenotrophomonas
Chryseobacterium
Brevibacillus
Pseudomonas
Unclassified
Delftia
Paenibacillus
Genus
B.
1115
1102
567
523
393
199
117
87
59
47
43
40
38
36
36
35
33
30
26
24
22
22
22
21
21
21
20
19
19
18
18
16
15
14
12
11
11
11
10
10
US
TJ
SE
RU
RO
PT
PL
NL
LI
IT
PK
GB
FR
FI
ES
EE
DE
CZ
CH
BY
CA
BE
AZ
AT
1500
1000
500
0
ASVs Per Sample

Figure EV2. (**A**) Relative abundance of top 10 genera across all Arabidopsis accessions. (**B**) Presence of shared and unique genera of Arabidopsis seed microbiome across all accessions.

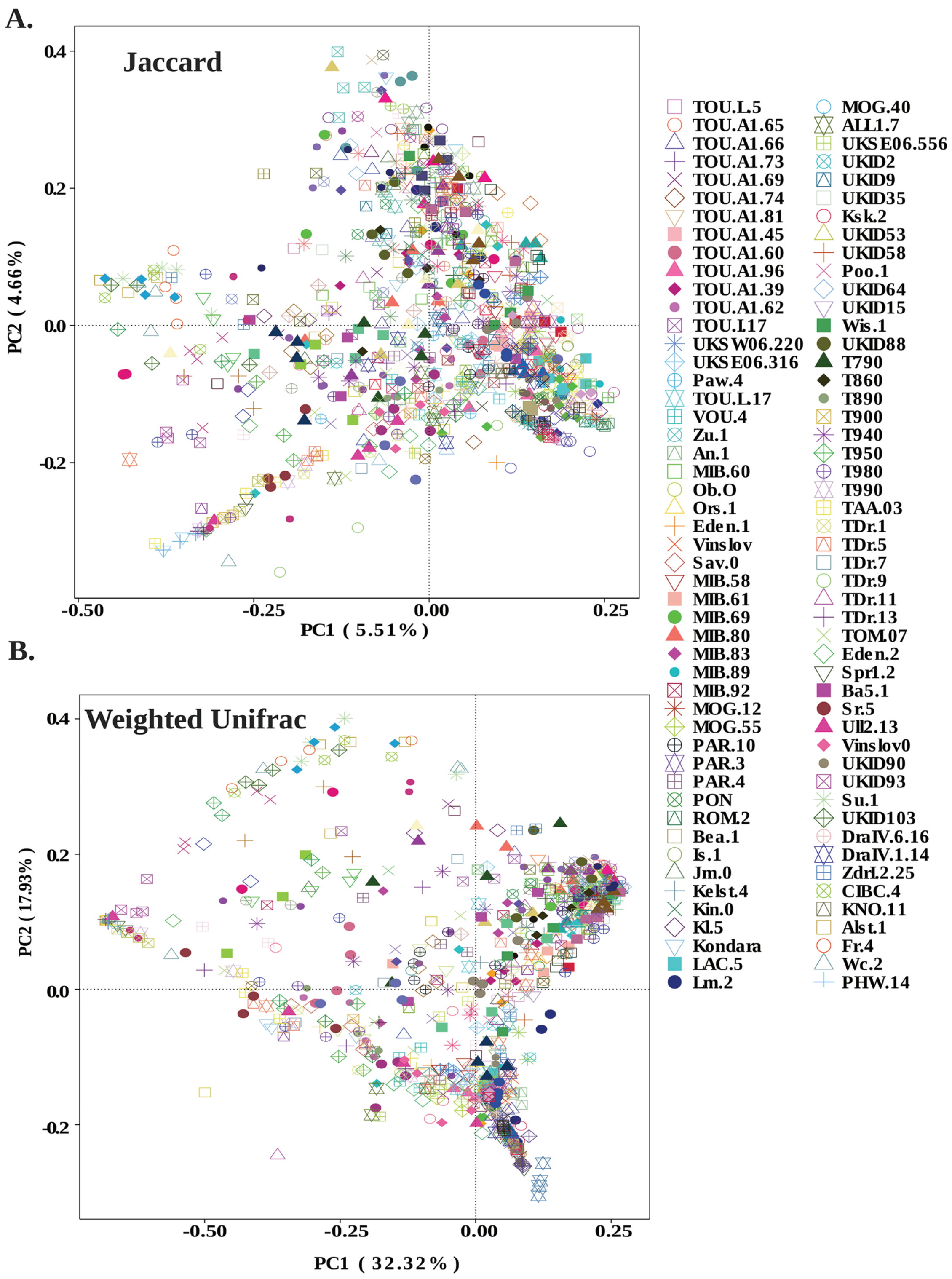

Figure EV3. PCoA plot of beta diversity of Arabidopsis seed microbiome.

Plots by Jaccard (A) and (B) Weighted Unifrac diversity measures.

© The Author(s)

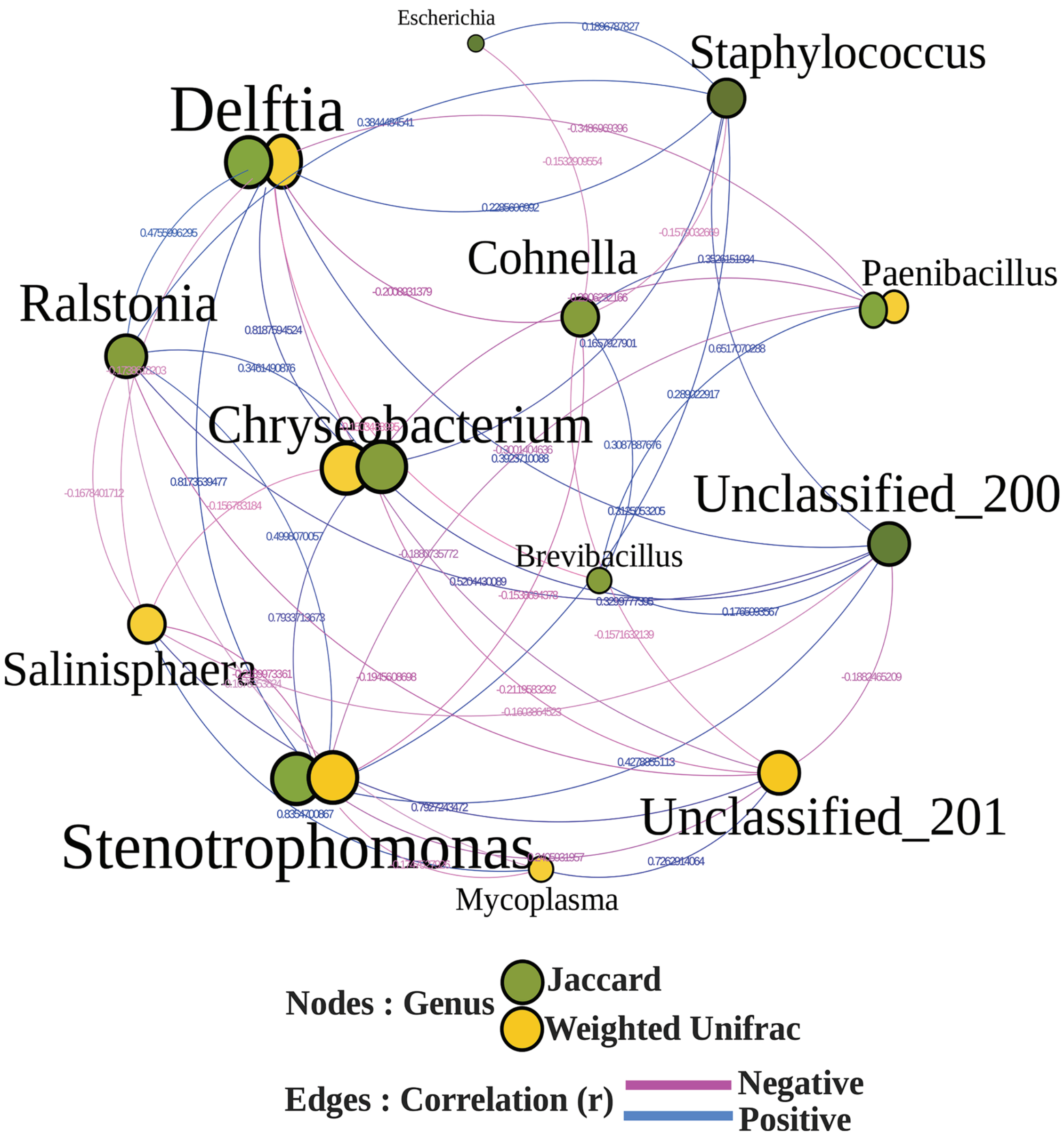

Figure EV4. Network of genus abundance.

The network shows the correlation patterns among genera in relation to Jaccard and Weighted UniFrac PC1 diversity measures. The color of the nodes represents their correlation occurrence with the diversity measures, while the node size corresponds to the degree of connectivity. Edges depict the Spearman correlation values, with positive correlations highlighted in blue and negative correlations in magenta.

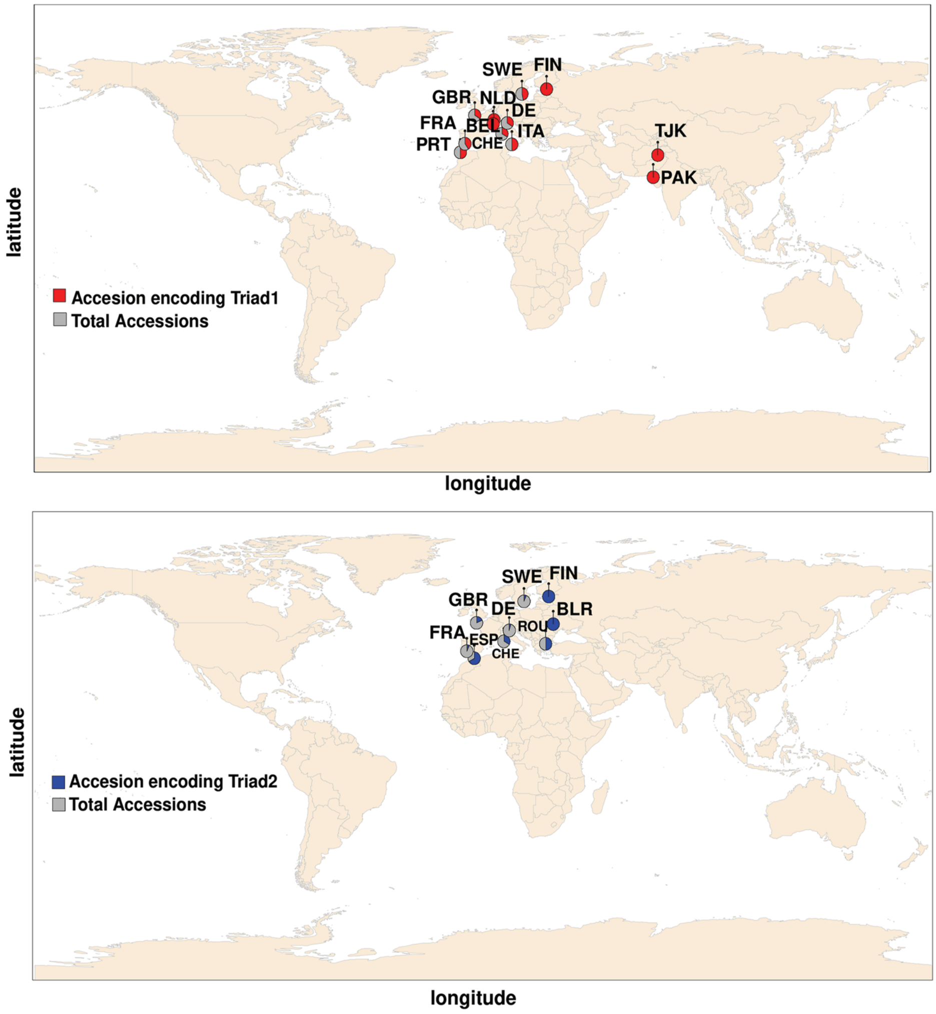

Figure EV5. Accessions encoded by triads across different countries.

Triad 1 includes *Chryseobacterium, Delftia, Stenotrophomonas* and Triad 2 *Paenibacillus, Brevibacillus and Cohnella*. We generated pie charts overlaid on a world map to represent each country's contribution to the total accessions versus those containing triads, defined by the presence across all genus of respective triad.

 © The Author(s)
